# Effects of sphingolipid metabolism disorders on endothelial cells

**DOI:** 10.1186/s12944-022-01701-2

**Published:** 2022-10-13

**Authors:** Yali Lai, Yue Tian, Xintong You, Jiangnan Du, Jianmei Huang

**Affiliations:** School of Traditional Chinese Materia Medica, Beijing University of Chinese Medicine, Beijing, China

**Keywords:** Sphingolipid metabolism, Endothelial cells, Sphingosine-1-phosphate, Sphingosine kinase, Ceramide, Serine, Ceramide kinase, Sphingosine-1-phosphate lyase, Cardiovascular disease

## Abstract

Many cardiovascular disorders, including atherosclerosis, hypertension, coronary heart disease, diabetes, etc., are characterized by endothelial cell dysfunction. Endothelial cell function is closely related to sphingolipid metabolism, and normal sphingolipid metabolism is critical for maintaining endothelial cell homeostasis. Sphingolipid metabolites or key enzymes in abnormal situation, including sphingosine, ceramide (Cer), sphingosine-1-phosphate (S1P), serine, sphingosine kinase (SPHK), ceramide kinase (Cerk), sphingosine-1-phosphate lyase (S1PL) etc., may have a protective or damaging effect on the function of endothelial cells. This review summarizes the effects of sphingolipid metabolites and key enzymes disordering in sphingolipid metabolism on endothelial cells, offering some insights into further research on the pathogenesis of cardiovascular diseases and corresponding therapeutic targets.

## Introduction

### Sphingolipids and sphingolipid metabolism

**Fig. 1:**
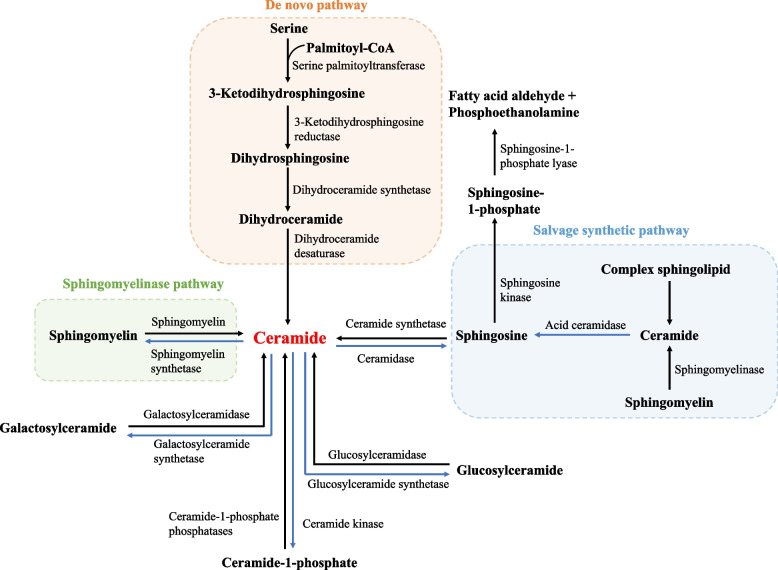
Sphingolipid metabolic pathway (black arrow represents anabolic pathway of Cer and blue arrow represents catabolic pathway of Cer)

Sphingolipids including sphingosine, ceramide (Cer), sphingosine-1-phosphate (S1P), sphingomyelin and glycosphingolipids, are essential parts of cell membranes and organelles that maintain cellular homeostasis. Besides, some sphingolipids, such as S1P and Cer, act as important second signaling molecules that regulate the processes of cell growth, differentiation, senescence and programmed cell death, performing a variety of biological functions1 [1]. In addition to performing the above functions to keep endothelial cells in their normal state, sphingolipids at normal levels also give endothelial cells the required conditions to keep their normal vascular tone, vascular permeability, oxidative stress response, etc. Once sphingolipid metabolism is disrupted, endothelial cells are inevitably harmed, which eventually lead to a range of cardiovascular disorders. For instance, S1P will damage the endothelial barrier to some extent and promote atherosclerosis [2, 3]; Cer will induce oxidative stress and apoptosis of endothelial cells, which also breaks the homeostasis of vascular endothelium, aggravating cardiovascular diseases such as atherosclerosis and heart failure [4, 5]; abnormal rise of sphingomyelin and glycosphingolipids will aggravate atherosclerosis [6, 7]. It can be seen that sphingolipid metabolism is important for endothelial cells to perform their normal physiological functions, so it's critical to investigate the relationship between sphingolipid metabolism and endothelial cells. Cer is the core of sphingolipid metabolism. The entire sphingolipid metabolic pathway unfolds around the synthesis and catabolism of Cer, as well as complex sphingolipid synthesis from Cer [1] (Fig. 1). The de novo synthesis pathway, the sphingomyelinase pathway, and the salvage synthesis pathway are the three primary pathways for the synthesis of Cer. The de novo synthesis pathway occurs in the endoplasmic reticulum, where L-serine and palmitoyl-CoA are successively catalyzed by three enzymes and converted to dihydroceramide, the latter of which is then dehydrogenated to Cer. The sphingomyelinase pathway mainly occurs in the cell membrane structure, where sphingomyelin is decomposed to Cer by the hydrolysis of acidic or neutral sphingomyelinas. The salvage synthesis pathway mainly occurs in lysosomes or late endosomes, where some complex sphingolipids (e.g., galactosylceramide, glucosylceramide, etc.) is degraded to Cer. There are several catabolism metabolic pathways of Cer: it can be phosphorylated to ceramide 1-phosphate or decomposited to sphingosine, the latter of which can be phosphorylated to S1P by SPHK; also, it can be converted to sphingomyelin or complex sphingomyelin (e.g., galactosylceramide, glucosylceramide, etc.).

The majority of earlier investigations on the dysregulation of sphingolipid metabolism focused on elucidating Cer and S1P, concerning their physiopathological effects and relevant mechanisms in the cardiovascular system. However, no comprehensive researches or reviews have been published on the effects of aberrant sphingolipid metabolism on endothelial cells. In this review, we summarize relevant researches during the past decade about the effects on endothelial cells caused by aberrant metabolites or key enzymes in sphingolipid metabolism, with the attention shifting the macroscopic cardiovascular system to the more microscopic endothelium., In addition to Cer and S1P, we mention the effects on endothelial cells when the levels of other sphingolipid metabolites or key enzymes are aberrant, containing serine, SPHK, ceramide kinase (CerK) and S1P lyase (S1PL).

### Endothelial cells and their physiological functions

Endothelial cells are a continuous monolayer of flat squamous epithelial cells, arranging on the inner surface of blood vessels and lymphatic vessels. Endothelial cells form a barrier between blood vessels and tissues, which covers the entire inner surface of blood vessels. On the one hand, endothelial cells play a role of static semipermeable membrane to regulate the selective exchange of substances between blood and tissue fluid. On the other hand, endothelial cells have the functions of secretion, metabolism and immunity [8], mediating vascular permeability, vasomotion, stress response, pathological vascular regeneration and inflammatory response [9]. Clinically, endothelial cell damage and vascular dysfunction are the main pathological features of typical cardiovascular diseases such as atherosclerosis, hypertension, coronary heart disease, and diabetes [10–14], indicating that endothelial cell dysfunction is closely related to the occurrence of these cardiovascular diseases. Therefore, clarifying the effects of sphingolipid metabolism disorders on endothelial cell function can provide a theoretical foundation for further research into the cardiovascular diseases’ mechanism and therapeutic approaches.

### Relationship between sphingolipid metabolism disorders and cardiovascular diseases

In a variety of cardiovascular diseases (e.g., atherosclerosis, hypertension, coronary heart disease, arrhythmia, heart failure, etc.), sphingolipid molecules at abnormal levels could be detected [15], suggesting that there is certain relationship between sphingolipid metabolism disorders and cardiovascular diseases. This founding is of great significance for the diagnosis and treatment of cardiovascular diseases.

Previous studies on sphingolipids associated with cardiovascular disease has focused mostly on Cer and S1P, with little attention paid to other sphingolipid molecules. Cer has several subtypes and their functions are not identical: short-chain ceramide induces oxidative stress, long-chain ceramide has strong cytotoxicity, and ultra-long-chain ceramide is related to insulin resistance; these functions are linked to the occurrence and development of atherosclerosis [5]. Moreover, Cer has a positively correlation with heart failure [5]. S1P exerts anti-atherosclerotic effects when it combines with high density lipoprotein (HDL) [16] or mediates phosphatidylinositol-3-kinases (PI3K)/protein serine-threonine kinase (AKT) signaling pathway [17]. Nevertheless, it is undeniable that S1P combining with albumin plays an important role in the progress of atherosclerosis by mediating S1PR2 [2, 3]. S1P participates in the process of myocardial fibrosis, cardiac remodeling, heart failure, pulmonary hypertension and the development of arrhythmias. Other sphingolipid molecules such as sphingomyelin and glycosphingolipids also have correlation with cardiovascular diseases. For example, sphingomyelin and glycosphingolipids play regulatory roles in atherosclerosis [15]. When sphingomyelin synthetase is overexpressed, the plasma sphingomyelin level will increase abnormally, which will aggravate atherosclerosis [7]. Unfortunately, it is yet unknown how sphingomyelin affects blood pressure, heart rhythm and cardiac function [15]. When investigating hyperglycemia induced atherosclerosis in mice based on untargeted plasma metabolomics, Dang [6] et al. found that glycosphingolipids were positively associated with atherosclerosis induced by hyperglycemia.

According the information above, there is a close relationship between abnormal sphingolipid metabolism and cardiovascular disease: aberrantly elevated Cer levels exacerbate atherosclerosis and heart failure; abnormal S1P levels have bidirectional effects on atherosclerosis, and exacerbate fibrotic processes, cardiac remodeling, heart failure, pulmonary hypertension and arrhythmias; aberrantly elevated levels of sphingomyelin and glycosphingolipids aggravate atherosclerosis. Since dysfunction of endothelial cells is a general phenomenon in cardiovascular diseases [18], it is critical to explore the effects of sphingolipid metabolism disorders on endothelial cells. Herein we comprehensively sort out and analyze the effects on endothelial cells when important sphingolipid molecules and key enzymes are in disorder, in order to provide a certain theoretical basis for further exploration of the relationship between sphingolipid metabolism and endothelial cells, and help better clarify the pathogenesis of cardiovascular disease based on sphingolipid metabolism disorders.

#### Bidirectional effect of S1P on endothelial cells

S1P is a bioactive sphingolipid metabolite which is widely expressed in the human body and is mainly derived from the phosphorylation of sphingosine in the sphingolipid metabolic pathway. S1P binds to the different sites of sphingosine-1-phosphate receptor (S1PR), regulating the growth state of endothelial cells and mediating multiple signaling pathways to engage in the physiopathological processes of the cardiovascular system [19].

#### Protective effects

In plasma, 65% S1P binds to HDL, 30% to albumin, and the remaining amounts to other lipoproteins [2]. Apolipoprotein M (ApoM), a minor apolipoprotein on HDL, is the S1P carrier on HDL, in this situation, ApoM and S1P exist in the form of a complex [20].The complex is a key component of HDL, which regulates adhesion molecule abundance, leukocyte-endothelial adhesion and endothelial barrier in the endothelium [21]. There are five S1P receptors, S1PR1, S1PR2, S1PR3, S1PR4 and S1PR5. S1P bound to HDL can protect endothelial cells via promoting endothelial cell survival and preventing their apoptosis through S1PR1, S1PR2 and S1PR3. Mario Ruiz et al. [21] showed that the complex of ApoM and S1P can restrict monocyte adhesion to the endothelium by binding to S1PR1 and preserve the integrity of the endothelial barrier under inflammatory conditions. Under the S1PR1/PI3K pathway, S1P can protect the function of the endothelial barrier through the spread of endothelial cells [22], the stabilization of endothelial cell–cell junctions [23] and NO-mediated inhibition of endothelial cell contraction [24]. Yang Liu et al. [25] found that the complex of ApoM and S1P could activate the PI3K/AKT pathway after binding to S1PR2, which will weaken the expression of pyroptosis-related proteins, inflammatory cytokines and adhesion molecules while enhancing the phosphorylation of PI3K and AKT in endothelial cells induced by tumor necrosis factor (TNF)-α, thereby reducing injury and inflammation in human umbilical vein endothelial cells (HUVECs) induced by TNF-α. Kimura Takao [26] found that S1P may act on ERK, PI3K/AKT and PI3K/Rac/p38 MAPK pathways in downstream through G-protein-coupled receptor EDG-1/S1PR1 and EDG-3/S1PR3, to mediate HDL-induced HUVECs migration and cytoprotection. Endothelial cells damage caused by intermittent hypoxia (IH) may be slowed by S1P-induced Akt/ endothelial nitric oxide synthase (eNOS) activation, so S1P might have a therapeutic potential for treating IH-induced endothelial cells damage [27]. Beibei Wang et al. [28] discovered that S1PR1 and endoglin (Eng) are both required for retinal angiogenesis in postnatal mice and that activation of S1PR1 or Eng increases vascular barrier function, implying that S1P controls vascular homeostasis by activating the S1PR1-Eng signaling pathway in endothelial cells. Additionally, Takeshi Nowatari et al. [29] discovered that human liver sinusoidal endothelial cells (LSECs) showed proliferative and anti-apoptotic effects after co-culturing with S1P: they found that S1P increased interleukins (IL)-6 and vascular endothelial growth factor (VEGF) production in LSECs, which in turn promoted hepatocyte proliferation; S1P induces LSECs proliferation by activating Akt and extracellular signal related kinase pathways; LSECs apoptosis was suppressed by changing the expression levels of oligomerization of pro-apoptotic B lymphocytoma-2 gene (Bcl-2), Bax and cleaved caspase-3.

Adenosine 5’-monophosphate-activated protein kinase (AMPK) is widely expressed in endothelial cells. In inflammatory situations, AMPK exerts anti-inflammatory and protective effects on the endothelium barrier, according to research conducted in vivo and in vitro [30]. Sophie Dennhardt et al. [31] used the electric cell-substrate impedance sensing (ECIS™) system to real-time monitor the endothelial barrier function in human microvascular endothelial cells (HMEC)-1 and murine glomerular endothelial cells (GENCs). Originally, they found that after disrupting the cell barrier with lipopolysaccharide (LPS)and TNF-α, the disrupted barrier in HMEC-1 was repaired to some extent, while the barrier in GENCs was still damaged. Afterwards, it was found that S1P could rapidly phosphorylate AMPK in HMEC-1, while S1P had no obvious barrier enhancement effect in HMEC-1 with small interfering RNA (siRNA)-mediated AMPK knockdown; at the meanwhile S1P could not phosphorylate AMPK in GENCs. It can be seen from the experimental results that S1P might play a role in promoting the barrier stabilization of HMEC-1 through the mechanism involving phosphorylation of AMPK, while AMPK in GENCs showed no barrier protection effect due to its inability to be activated by phosphorylation. Thus, it was also illustrated that, to some extent, S1P has selectivity on the phosphorylation of AMPK. What’s more, S1P may control the tyrosine phosphorylation of RacGTPase effector proteins PAK1 and VE-cadherin by mediating AMPK to protect pulmonary vascular endothelium, thus alleviating the LPS-induced lung dysfunction [32]. Xiaojing Sun et al. [33] found that S1P also enhanced glomerular endothelial cells activation mediated by anti-myeloperoxidase antibody-positive IgG. The above experiments verified that S1P has a protective effect on a variety of endothelial cells, which strongly proved the endothelial cell protective effect of S1P.

Endothelial progenitor cells (EPCs) are able to repair damaged blood vessels by proliferation and migration, and ultimately EPCs will differentiate into endothelial cells. S1P can promote the proliferation of EPCs and attenuate their apoptosis through the S1PR1/S1PR3/PI3K/AKT pathway [34], thereby promoting the differentiation of EPCs into endothelial cells, which also reflects the protective effect of S1P on endothelial cells. S1P can not only promote the differentiation of EPCs into endothelial cells, but also promote the differentiation of other stem cells into endothelial cells, such as bone marrow stem cells [35]. During the culture of primary mesenchymal stem cells (PR-MSCs), Lu W et al. [35] added S1P to it and found that the apoptotic rate of PR-MSCs cultured in hypoxia in the S1P-treated group was significantly lower than that in the control group. After culturing with VEGF for 7 days, endothelial cell-related genes were highly expressed in PR-MSCs treated with S1P compared with the control group, which was consistent with the enhancement of hepatocyte growth factor, stromal cell-derived factor-1 and insulin-like growth factor-1, indicating that S1P could improve the PR-MSCs differentiate into endothelial cells.

If inflammation occurs, S1P levels will rise locally, inducing endothelial adhesion molecules, recruiting inflammatory cells and activating dendritic cells. And at the same time, S1P can protect the endothelium through continuously sealing endothelial cell–cell contacts, reducing vascular leakage and inhibiting cytokine induced leukocyte adhesion [19], which finally exerts a protective effect on the endothelium.

#### Damaging effects

Kerage et al. [36] found that, physiologically, low concentrations (about 0.1 mmol/L) of S1P could maintain the endothelial barrier, while high concentrations (about 10 mmol/L) of S1P showed a damaging effect on the endothelial barrier and increased vascular permeability. S1P can inhibit the barrier function of endothelial cells through S1PR2/Rho/ROCK/PTEN pathway [37], S1PR2/PI3K/AKT pathway [38], S1PR2/AKT/eNOS pathway [38, 39], and RAGE-JAK2/STAT3 pathway [40].

Through a series of supporting experiments and information as follows, Weihua Liu [38] discovered that under high glucose condition, blocking the PI3K/AKT signaling pathway can partially mediate endothelial dysfunction when S1P binds to S1PR2. Under high glucose condition, S1PR2 is the main expression type of S1P receptor in human coronary artery endothelial cells (HCAECs). In HCAECs under high glucose and normal glucose conditions, NO level and eNOS activity significantly decreased after S1P treatment, whereas PMN adhesion, VCAM-1, and ICAM-1 protein levels considerably increased. After S1P is treated in HCAECs under high glucose and normal glucose conditions, NO levels and eNOS activity significantly decreased, while levels of PMN adhesion, VCAM-1 and ICAM-1 protein dramatically increased. However, blocking S1PR2 with the specific antagonist JTE-013 and siRNA led to the increase of NO levels and eNOS activity and the decrease of PMN adhesion, and lowered VCAM-1 and ICAM-1 protein levels induced by S1P. What’s more, in S1P treated cells under high glucose condition, S1PR2 blocking significantly increased the phosphorylation levels of PI3K and AKT, which could be inhibited by PI3K inhibitor wortmannin. The above results indicate that, under high glucose condition, S1P/S1PR2 mediates human coronary endothelial dysfunction partially by inhibiting the phosphorylation of PI3K/AKT or reducing NO levels and eNOs activity.

Weihua Liu and Shuangfeng Lin et al. [39] cultured human aortic endothelial cells with treatment of S1P, SPHK1 inhibitor and AKT inhibitor in high glucose condition, and some phenomena were observed as follows. Firstly, they found that S1P could significantly reduce the NO content in culture supernatant of endothelial cells induced by high glucose. Also, S1P could promote granulocyte-endothelial cell adhesion, considerably increase ICAM-1 protein expression in endothelial cells, and inhibit endothelial cell migration and AKT/eNOS signaling pathway activation. However, in high glucose-induced endothelial cells given with SPHK1 inhibitor, the production of S1P was reduced, along with marked improvement of the above functional indicators and restoration of the activated AKT/eNOS signaling pathway. Besides, Weihua Liu [38] found that S1PR2 was the main expression type of S1P receptor when studying the effect of S1P on HCAECs under high glucose condition. Based on the above results, it could be speculated that S1P may aggravate the dysfunction of human aortic endothelial cells cultured in high glucose condition by inhibiting the activation of S1PR2/AKT/eNOS signaling pathway.

Under hyperglycemia induction, oxidative stress response of endothelial cells enhanced, which caused the dysfunction of endothelial cells. Chen S et al. [41] exposed HUVECs to high concentrations of glucose and found that the expression levels of S1PR1 significantly reduced while the expression levels of S1PR2 significantly increased, accompanied with a significant increase in cellular dysfunction due to oxidative stress and hyperglycemia. In addition, they found that the effect of high glucose on S1PR1 and S1PR2 levels could be reversed through overexpression of S1PR1 caused by pAD-S1PR1 transfection or down-regulation of S1PR2 by shRNA, which alleviated oxidative stress and inhibited HUVECs dysfunction. A series of biological activities of endothelial cells mediated by S1PRs can be regulated by S1P, of which S1PR1 and S1PR2 play a key role in hyperglycemia-induced endothelial cell dysfunction. Therefore, S1P can mediate hyperglycemia-induced dysfunction of HUVECs through binding to S1PR1 and S1PR2.

Through literature mining technique, pathway enrichment analysis and MetPA analysis, Cong Zhang [40] screened S1P, a key metabolite with potentially important impacts on rat diabetes, and performed cell viability experiments by MTT assay, which showed that S1P would affect the viability of rat retinal microvascular endothelial cells (RMECs) and damage rat RMECs. Furthermore, the author conducted rat RMECs scratch assay to evaluate the migration ability of rat RMECs, which showed that S1P could reduce the migration ability of RMECs. The above two experiments both illustrate the damaging effect of S1P on rat RMECs.

Besides, Cong Zhang [40] detected MDA, SOD, intracellular reactive oxygen species content, apoptosis level and other indicators of RMECs. The experimental results consistently showed that S1P could aggravate the oxidative stress injury of RMECs induced by high glucose. Then, network pharmacology studies showed that S1P may induce a series of oxidative stress injuries such as increased reactive oxygen species (ROS) level and apoptosis of RMECs by activating RAGE-JAK2/STAT3 signaling pathway.

**Fig. 2:**
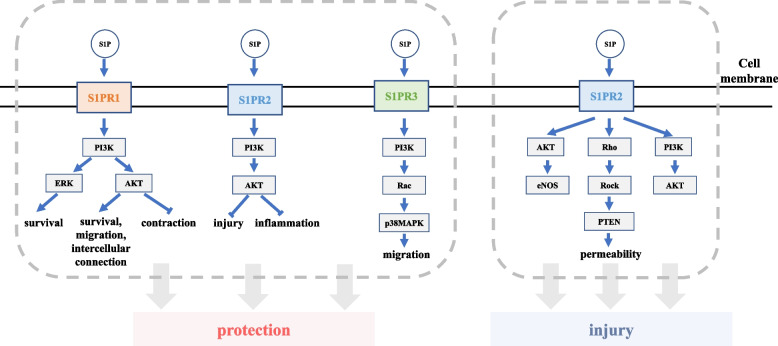
Signaling pathway of the protective or damaging effect of S1P on endothelial cells

Now we sort out the bidirectional effect of S1P on endothelial cells and the relevant signaling pathway mentioned in this review (Fig. 2). It can be seen that S1P can play the opposite role after binding to the same receptor (S1PR2), but the current research can not systematically clarify its specific mechanism. Apart from the signaling pathways mentioned in Fig. 2, there are possibly more signaling pathways of S1P’s effects on endothelial cells. So, more details of this extent are needed to provide more theoretical basis for further research of cardiovascular disease.

## Effects of SPHK on endothelial cells

SPHK, one of key enzymes regulating sphingolipid metabolism, catalyzes the phosphorylation of dihydrosphingosine to S1P in sphingolipid metabolic pathways, playing relevant biological roles by regulating the metabolism of the catalytic product S1P. In mammalian cells, SPHK mainly includes two subtypes, SPHK1 and SPHK2. Although two subtypes of SPHK catalyzes similar biochemical reactions, their biological roles are quite different. Previous studies have shown that SPHK1 mainly promotes cell survival and proliferation, while SPHK2 is mainly related to apoptosis [42]. The biological effects exerted by SPHK on endothelial cells include the perspectives of endothelial cell permeability, proliferation and migration.

### Effects on permeability of endothelial cell

#### Increase permeability of endothelial cells

Yi Wanhua et al. [43] discovered that the activation of SPHK1 could increase the permeability of lung microvascular endothelial cells (PMVECs) hit by heat combined with endotoxin, and S1PR3 may be the downstream S1P receptor that is mainly involved. If the expression of SPHK1 is specifically inhibited, the damage of PMVECs can be reduced.

Itagaki K et al. [44] monitored the permeability of human pulmonary artery endothelial cells and human pulmonary microvascular endothelial cell in real time after exposure to activated neutrophils (PMNs), finding that the permeability of endothelial cells had increased after being stimulated by activated PMNs, and this increase in permeability could be attenuated by co-cultures exposured to SPHK inhibitors (SKI-2, N, N-dimethylsphingosine [DMS]) or Ca^2+^ entry inhibitors (Gd^3+^, MRS-1845). It was indicated that there is a close link between SPHK and increased permeability of endothelial cell induced by activated PMNs.

#### Decrease permeability of endothelial cells

Sphingosine phosphorylation is induced by SPHK to produce S1P, which stabilizes inter endothelial connections and inhibits microvascular leakage. Tauseef M et al. [45] confirmed that SPHK1 plays an indispensable role in mediating the reversal process of increased permeability of mice PMVECs. Meanwhile, it was found that SPHK1-S1P-S1PR1 signaling pathway activation enhances the homeostasis of endothelial barrier which is injured by endothelial inflammatory mediators, and activation of SPHK1 has a preventive effect on pulmonary edema induced by lipopolysaccharide or thrombin-binding protease-activated receptor-1 (PAR-1). In fact, the process of S1P production caused by up-regulated SPHK1 activity is a negative feedback mechanism to limit the increase of endothelial permeability caused by different inflammatory stimuli, which indicates that SPHK1 has an important role in anti-inflammatory.

By analyzing the above studies concerning the effect of SPHK on endothelial cell permeability, it can be found that SPHK1 is an important subtype affecting endothelial cell permeability. But intriguingly, the same subtype of SPHK has two opposing effects on the permeability of endothelial cells, which may be related to the downstream S1P receptor. According to the current studies, it is preliminarily speculated that endothelial cell permeability can be enhanced when S1P’s downstream receptor is S1PR3, while it can be reduced when downstream receptor is S1PR1. Yet further exploration and verification are needed in search for the specific mechanism of its dual regulation of endothelial cell permeability.

#### Effects on proliferation and migration of endothelial cells

Reduced SPHK1 expression can greatly restrict HUVECs proliferation and migration, while its catalytic product S1P can increase them [46]. Ginsenoside K can inhibit the production of S1P, SPHK1 activity and SPHK1 expression in HUVEC, affect the sphingolipid rheostat, and inhibit HUVECs migration and angiogenesis by reducing the expression of metalloproteinase (MMP), according to a study on ginsenoside K's ability to inhibit angiogenesis [47].

Vascular endothelial growth factor (VEGF) is a crucial growth factor that can promote the proliferation of endothelial cells, angiogenesis and other activities. It was reported that the levels of VEGF receptors were significantly higher in various pathological conditions, such as tumor development and vascular injury, indicating that VEGF has a promoting effect on the division and proliferation of endothelial cells; additionally, VEGF mediated by protein kinase C could induce endothelial cell proliferation, angiogenesis and repair through the activation of SPHK [42]. Hepatocyte growth factor (HGF) can also induce the migration of endothelial cells and play a crucial role in angiogenesis [48]. Jun Yi et al. [49] proposed that HGF could induce endothelial cell migration through the SPHK pathway: expression of wild-type SPHK significantly enhanced intracellular SPHK activity and promoted HGF-induced endothelial cell migration; while SPHK negative dominant gene significantly inhibits HGF-induced endothelial cell migration.

It is known that LIM-domain-only 2 (Lmo2) transcription factor is involved in hematopoiesis and vascular remodeling. Matrone G et al. [50] found that if Lmo2 gene was knocked out, the interaction between Lmo2-SPHK1 genes was weakened, the formation of intersegmental vessels was impaired, and cell migration was inhibited; it was also found that Lmo2 was necessary for SPHK1 gene expression in endothelial cells, and SPHK1 was the downstream effector of Lmo2. Thereby we can know that Lmo2 regulates SPHK1 and promotes endothelial cell migration.

Schwalm S et al. [51] found that NO donors could specifically up-regulate the expression and activity of SPHK1 in human endothelial cells, which in turn significantly contributed to the migration ability and tube formation of endothelial cells, indicating that SPHK1 may make a difference in the migration and proliferation of endothelial cells.

The above experiments all demonstrated that SPHK had a promoting effect on proliferation or migration of endothelial cells. In addition, with the continuous development of studies on cell biological activity affected by sphingolipid metabolism, researchers have proposed the concept of “sphingolipid rheostat”. SPHK can regulate the relative levels of S1P and Cer through the function of “sphingolipid rheostat”, which in turn makes a dual influence to the apoptosis and proliferation of endothelial cells and ultimately determines the fate of endothelial cell survival or death [52].

#### Other effects

SPHK1 regulates vasoconstriction and vasorelaxation and is essential in vasorelaxant effects mediated by angiotensin II. Production of NO is significant decreased if endothelial cells are exposed to SPHK1 inhibitors, which causes endothelium-dependent vasoconstrictor induced by responses angiotensin II [42]. In addition, forced expression of SPHK1 in resistance arteries enhances vasoconstriction, while expression of the negative dominant SPHK1 leads to vasorelaxation [53], reflecting the bidirectional regulation of vascular tension by SPHK1.

When the body is in pathological conditions such as ischemia, SPHK can alleviate the oxidative stress of vascular endothelial cells by inducing angiogenesis, regulating vasoconstriction and vasorelaxation and improving the stress tolerance of vascular endothelial cells, thereby maintaining the integrity of blood vessels [42].

Down-regulation of SPHK and MAB S1PR1 and S1PR3 impacted intercellular communication between human mesenchymal progenitor mesoangioblasts and human mammary vascular endothelial cells, drastically lowering in vivo angiogenesis as measured by the Matrigel plug experiment [54]. This illustrates the significant attenuation of angiogenesis in vivo when SPHK is inhibited.

Although EPCs have been demonstrated to play a role in the growth and repair process of blood vessels, including wound healing, limb ischemia, myocardial infarction and tumor angiogenesis [55–57], their application in vascular treatment has been restricted due to their low presence in bone marrow and peripheral blood. Existing studies have shown that overexpression of SPHK1 can promote the dedifferentiation of mature human endothelial cells into a progenitor phenotype and increase the number of EPCs, which provides a new idea for the treatment of cardiovascular diseases [58].

#### Effects of Cer on endothelial cells

As the core of sphingolipid metabolic pathway, Cer plays an important part in the sphingolipid metabolic pathway. Like other sphingolipid metabolites, Cer is not only an important component of cell membrane and organelle membrane, but also has a certain effect on cell function by participating in signal transduction in the process of cell growth and apoptosis. Additionally, Cer is currently the primary research direction focusing on sphingolipids related cardiovascular illness, and it has been established that Cer has certain associations with vascular-related diseases such hypertension, atherosclerosis, angiogenesis and remodeling [9]. The relevant effects of Cer on the biological function of endothelial cells are as follows.

#### Increase oxidative stress response of endothelial cells

Cer triggers the formation of ROS, which in turn activates different ceramide synthesis pathways. Oxidative stress responses are continuously induced in endothelial cells via this positive feedback, ultimately mediating endothelial cell function [59]. It has been demonstrated that Cer-rich lipid rafts accumulate in endothelial cells under stimuli such as Fas ligand and TNF-α, thereby enhancing the activity of adenine dinucleotide phosphate (NADPH) oxidase and promoting generation of ROS, explaining the specific mechanism of Cer-induced oxidative stress response in endothelial cells [60].

#### Mediate senescence and apoptosis of endothelial cells

Venable ME et al. [61] cultured HUVECs to different population doubling levels and measured Cer levels, finding that endogenous Cer levels increased 2.4-fold with aging; after long-term treatment of lower passage cells with exogenous c6-Cer, it was found that cell proliferation and DNA replication were inhibited, and the expression of β-galactosidase associated with aging increased, indicating that this treatment induced an aging phenotype. Thus, it implies that Cer may be a general mediator of cellular senescence.

Numerous investigations have supported the function of Cer in mediating endothelial cell apoptosis. Colin Niaudet al. [62] found that ionizing radiation and anisomycin induced p38 mitogen-activated protein kinase (MAPK)-dependent apoptosis in endothelial cells, and this activation was dependent on the generation of acid sphingomyelinase and Cer, verifying that Cer has a role in driving endothelial cell apoptosis. In addition, many factors such as septic shock, heat shock, LPS, ultraviolet light, ionizing radiation, oxidative stress and other cellular stressors can activate acid sphingomyelinase, thereby stimulating the production of Cer and promoting endothelial cell apoptosis [63].

**Fig. 3:**
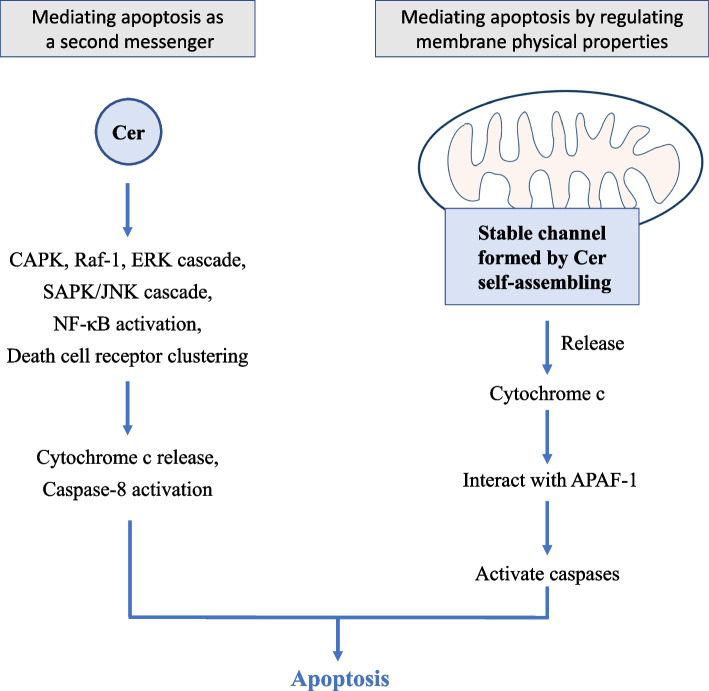
Apoptosis of endothelial cells induced by Cer

Cer can mediate endothelial cell apoptosis through several pathways (Fig. 3). Cer can act directly with specific protein domains as a second messenger/bioactive molecule, so as to play a corresponding role in promoting apoptosis. For example, Cer can stimulate cAMP dependent protein kinase (CAPK), Ras associated factor-1 (Raf-1), ERK cascade, SAPK/JNK cascade, NF-κB activation and death cell receptor aggregation, inducing the release of mitochondrial cytochrome C or the activation of Caspase-8, thereby promoting apoptosis [64]. Moreover, via promoting the Bcl-2 family proteins, Cer can also reduce the expression of anti-apoptotic Bcl-2 gene and mediate endothelial cell apoptosis [64]. In addition to its pro-apoptotic role as a second messenger, Cer can also promote apoptosis by regulating the physical the membrane properties. Cer prompts apoptosis by self-assembling on the mitochondrial outer membrane to form large stable channels that can release cytochrome C, further interacting with apoptotic protease activating factor 1 (APAF-1) to activate a variety of caspases [63] and finally driving the apoptosis. Moreover, a saturated free fatty acid, palmitic acid can activate phosphatase through Cer, inducing endothelial VEGF resistance and inhibiting angiogenesis, which indicates that Cer has a damaging effect on angiogenesis and drives apoptosis [65].

#### Regulation of vascular tension and blood pressure

NO, synthesized from L-arginine catalyzed by eNOS in endothelial cells, is one of the most important substances affecting vascular tone. Anne et al. [66] knocked out the mice endothelial cell specific serine palmitoyl transferase long chain subunit 2 (Sptlc2) and found that the de novo Cer synthesis pathway in Sptlc2 knockout mice was inhibited, NO release was increased, eNOS activity was enhanced in the mesenteric artery, and blood pressure was decreased. This study demonstrated that Cer could inhibit vasodilation and increase blood pressure by inhibiting NO release in endothelial cells. Many studies have shown that Cer can inhibit vasodilation by inhibiting the synthesis of NO or promoting the conversion and breakdown of NO in endothelial cells [9]. In addition to inhibiting NO release, Cer can also increase the stiffness of cell membranes through intermolecular hydrogen bonds, reducing the fluidity of cell membranes and promoting vasoconstriction [67].

Additionally, it has been demonstrated that Cer inhibits the Akt/eNOS signaling pathway in HUVECs induced by high glucose [68]: high glucose levels in HUVECs also cause Cer accumulation in a dose- and time-dependent manner, which prevents insulin-mediated activation of Akt/eNOS signaling and subsequent NO production, thereby promoting vasoconstriction.

#### Effect of CerK/C1P pathway on endothelial cells

One of the key enzymes, ceramide kinase (CerK), catalyzes the conversion of Cer into ceramide-1-phosphate (C1P), which is important for the regulation of sphingolipid metabolism. CerK controls the growth, survival and migration of endothelial cells [69]. The degradation of endogenous C1P causes the Cer level increase and then mediates the apoptosis of endothelial cells [70].

C1P induces neointima formation by affecting cell proliferation and cell cycle upstream of ERK1/2 in vascular smooth muscle cells [71]. The CerK/C1P pathway might be a separate mechanism from the known VEGF pathway. Niwa et al. [72] added VEGF, fibroblast growth factor (FGF), TNF-α and other potent proangiogenic factors to dermal micro endothelial cells (DMECs) in the skin of CerK knockout rats, all failed to reverse the damage of DMECs, demonstrating that this pathway may have effects on vascular endothelial cell growth through other pathways.

#### Barrier effects of S1PL on endothelial cells

Under the effect of S1P lyase (S1PL), S1P is converted to fatty acid aldehyde and phosphoethanolamine. S1PL exerts vasoprotective effect on brain tissue endothelium under inflammatory conditions [73]. Cells with stable downregulation of S1PL have a more unstable barrier when not stimulated by inflammation. Whereas cells with stable downregulation of S1PL provided protection against the barrier breakdown caused by proinflammatory cytokine in an inflamed environment. Experiments have shown that cells with stable downregulation of S1PL can lead to a substantial accumulation of intracellular S1P and dihydro sphingosine 1-phosphate as well as various other molecular factors that may contribute to the protective barrier. This suggests that, SPL modulation is an effective way to suppress inflammatory responses and enhance barrier integrity in brain endothelial cells. Cells with stable downregulation of S1PL exhibited a strong downregulation of the cytokine IL-6, all of which contribute to their protective effects under inflammatory conditions.

#### Effects of serine on endothelial cells

When Vandekeere S et al. [74] explored the effect of phosphoglycerate dehydrogenase (PHGDH) on endothelial cells in the serine synthesis pathway, they found that serine synthesis in endothelial cells of PHGDH knockout mice was reduced, resulting in nucleotide synthesis damage, the reduction of GSH, NADPH and heme synthesis level, causing mitochondrial oxidative stress damage and promoting mitochondrial apoptosis, which thereby drove the endothelial cell apoptosis. It can be speculated from the above studies that serine has a certain protective effect on endothelial cells, and when a series of factors inhibit serine synthesis appear, the synthesis level of serine decreases and then promotes apoptosis of endothelial cell.

With MTT assay and commercial immunoassay methods, it was found that L-serine has antioxidant and cytoprotective effects through the elevation of some crucial antioxidant factors such as nuclear factor-erythroid 2 (NF-E2)-related factor 2 (Nrf2), heme oxygenase-1 (HO-1) and total Nitric Oxide (NOx) [75]. This suggests that the anti-atherosclerotic effect of L-serine may be achieved through the protection of endothelial cells as well as antioxidation.

## Summary and prospect

In summary, sphingolipid metabolites and related enzymes are closely associated to the proliferation, apoptosis, aging, migration, oxidative stress and other activities of endothelial cells, and affect the regulatory function of endothelial cells on vascular permeability, vasoconstriction and vasodilation, stress response, vascular regeneration and inflammatory response, etc. Besides this, sphingolipid metabolites also have a direct regulatory effect on cardiovascular function. Therefore, in-depth study of the relationship between sphingolipid metabolism and endothelial cells can provide reference for exploring vascular-related disease markers and finding corresponding therapeutic targets, and also provides clues for the pathogenesis of a series of diseases related to endothelial cell function.

## Data Availability

Not Applicable.

## Abbreviations

Cer: Ceramide
S1P: Sphingosine-1-phosphate
SPHK: Sphingosine kinase
HDL: High density lipoprotein
PI3K: Phosphatidylinositol-3-kinases
AKT: Protein serine-threonine kinase
S1PR: Sphingosine-1-phosphate receptor
ApoM: Apolipoprotein M
LPS: Lipopolysaccharide
TNF: Tumor necrosis factor
HUVECs: Human umbilical vein endothelial cells
APMK: Adenosine 5’-monophosphate-activated protein kinase
ECIS™: Electric cell-substrate impedance sensing
HMEC: Human microvascular endothelial cells
GENCs: Glomerular endothelial cells
EPCs: Endothelial progenitor cells
PR-MSCs: Primary mesenchymal stem cells
HCAECs: Human coronary artery endothelial cells
siRNA: Small interfering RNA
RMECs: Retinal microvascular endothelial cells
PMVECs: Pulmonary microvascular endothelial cells
PMNs: Neutrophils
PAR-1: Protease-activated receptor-1
VEGF: Vascular endothelial growth factor
HGF: Hepatocyte growth factor
Lmo2: LIM-domain-only 2
ROS: Reactive oxygen species
NADPH: Adenine dinucleotide phosphate
MAPK: Mitogen-activated protein kinase
CAPK: CAMP dependent protein kinase
Raf-1: Ras associated factor-1
Bcl-2: Pro-apoptotic B lymphocytoma-2 gene
APAF-1: Apoptotic protease activating factor 1
eNOS: Endothelial nitric oxide synthase
Sptlc2: Serine palmitoyltransferase long chain subunit 2
PHGDH: Phosphoglycerate dehydrogenase
CerK: Ceramide kinase
C1P: Ceramide-1-phosphate
FGF: Fibroblast growth factor
DMECs: Dermal micro endothelial cells
S1PL: Sphingosine-1-phosphate lyase
Nrf2: Nuclear factor-erythroid 2 (NF-E2)-related factor 2
HO-1: Heme oxygenase-1
NOx: Total Nitric Oxide
